# Exploring the Longitudinal Association Between Visual Function and Memory Performance Among Older Adults

**DOI:** 10.1093/geroni/igaf122.1621

**Published:** 2025-12-31

**Authors:** Jonathan Thomas, Shu Xu, Joshua Ehrlich

**Affiliations:** Texas A&M University Health Science Center, Bryan, Texas, United States; University of Michigan, Ann Arbor, Michigan, United States; University of Michigan, Ann Arbor, Michigan, United States

## Abstract

Older adults with a visual impairment (VI) are more likely to experience cognitive decline; however, limited longitudinal studies exist on how changes of specific visual function influence the trajectories of cognitive performance, including memory. Therefore, the purpose of this study is to examine the longitudinal association between visual function measures and memory performance among U.S. older adults. We analyzed three-year longitudinal data (2021-2023) from Medicare beneficiaries ≥ 71 years in the National Health and Aging Trends Study. Visual function was assessed based on presenting binocular near visual acuity [NVA], distance visual acuity [DVA], and contrast sensitivity [CS]. Any VI was defined as either near or distance VI [>0.30 logMAR], or CS impairment [< 1.55 logCS]. Memory performance was measured using immediate and delayed word recall, scored from 0-20, with higher scores indicating better memory. Longitudinal associations between visual function and memory were analyzed with growth curve model. Among 3,162 participants, 34% had any VI. Older adults with any VI (7.34) had lower baseline memory scores than those without any VI (9.19). Growth curve models showed significant between-person effects for all visual function measures (DVA: B=-2.73, p<.001; NVA: B=-2.89, p<.001; CS: B=-2.60, p<.001); however, only NVA (B=-1.16 p<.001) and CS (B=-0.77 p<.01) showed significant within-person effects. In conclusion, older US adults with worse overall visual function have lower memory performance, and a decline in NVA and CS over time is associated with a faster decline in memory performance. Findings highlight the importance of vision health in cognitive aging.

